# Efficacy and side effects of anti‐CD19 CAR T‐cell therapy in patients with relapsed/refractory gastrointestinal lymphoma

**DOI:** 10.1002/cam4.7064

**Published:** 2024-03-08

**Authors:** Yi Li Jiang, Juan Mu, Rui Cui, Xin Li, Jia Wang, Qing Li, Jingyi Li, Nan Mou, Qi Deng

**Affiliations:** ^1^ Department of Hematology, Tianjin First Central Hospital, School of Medicine Nankai University Tianjin China; ^2^ Shanghai Genbase Biotechnology Co., Ltd Shanghai China

**Keywords:** B‐cell lymphoma, chimeric antigen receptor (CAR), gastrointestinal lymphoma, relapsed/refractory

## Abstract

**Introduction:**

Although anti‐CD19 chimeric antigen receptor (CAR) T cell therapy was approved as a very effective salvage strategy in relapsed/refractory (R/R) B cell lymphoma, the experience in R/R gastrointestinal (GI) lymphoma is still insufficient.

**Methods:**

We summarized the efficacy and side effects of anti‐CD19 CAR T‐cell therapy in 12 patients with R/R GI lymphoma. Based on literature, the R/R GI lymphoma patients were divided into subgroups with different characteristics: Bulky/No bulky disease, Gastric/Gastrointestinal involvement, Gastrointestinal/Combined extra‐gastrointestinal lesions, Ulcer/Lumps or nodules type, With/without gastrointestinal bleeding.

**Results:**

The objective response rate (ORR) was 66.67% in these 12 patients. The ORR was 83.33% in no bulky disease group, 80.00% in gastric involvement group, 100.00% in ulcer type group, and 80.00% in no gastrointestinal bleeding group. The CR rate was 33.33% in these 12 patients. The CR was 50.0% in no bulky disease group, 60.00% in gastric involvement group, and 80.00% in ulcer type group. The PFS and OS rate of the 12 patients at 6 months after infusion were 54.55% and 58.33%, respectively. The overall survival (OS) at 6 months was higher in no bulky disease group. There was no difference of the OS or the progression free survival (PFS) at 6 months between the other groups. The mean peak of CAR‐T cells and Cytokine Release Syndrome (CRS) grade were higher in gastrointestinal lesions group. The mean peak of IFN‐γ and CRS grade were higher in gastrointestinal bleeding group. Four out of six patients in group of gastrointestinal lesions group were patient with high tumor burden. Patients with gastrointestinal involvement only were at higher risk for gastrointestinal bleeding.

**Conclusions:**

The ORR and CR of high tumor load, gastrointestinal involvement, lumps or nodules type and gastrointestinal bleeding group were lower. The CRS grade was higher in gastrointestinal lesions group and in gastrointestinal bleeding group. Patients with gastrointestinal involvement only were at higher risk for gastrointestinal bleeding.

## 1 INTRODUCTION

The gastrointestinal (GI) lymphoma is the most typical sites of primary extranodal non‐Hodgkin's lymphoma, accounting for about 30%–45% of all extranodal lymphomas, which is a malignant tumor originating from submucosal lymphoid tissue of gastrointestinal tract.^1^, ^2^, ^3^ The stomach is the most common involved site (60%–75%), followed by small intestine (20%–30%) in gastrointestinal tract.^4^, ^5^ B cell lymphoma, especially diffuse large B‐cell lymphoma (DLBCL), is the most common type in all GI lymphoma, whereas T cell lymphoma is less common.^5^, ^6^, ^7^ Patients with GI lymphoma might present with numbers of clinical manifestations known as gastrointestinal complications (GICs), including gastrointestinal bleeding, gastrointestinal obstruction, and gastrointestinal perforation.^8^ But there is insufficient of available data of GICs in GI lymphoma for clinical regimens.^9^, ^10^ The most common clinical manifestations were abdominal pain or discomfort, hematemesis or black stools, and changes in bowel habits.^11^

Anti‐CD20 antibody rituximab has improved the survival rate of patients with DLBCL in the past two decades.^12^ The treatment strategy for GI lymphoma has shifted away from surgery.^9^, ^13^ Combination therapy with rituximab significantly improved the efficacy and overall survival (OS) of patients with GI lymphoma.^14^, ^15^, ^16^ However, some patients do not respond to these combination therapies and become relapsed/refractory (R/R) GI lymphoma. Anti‐CD19 chimeric antigen receptor (CAR) T cell therapy was approved as a very effective salvage strategy in R/R DLBCL, mantle cell lymphoma, and follicular lymphoma.^17^, ^18^, ^19^, ^20^ However, the anti‐CD19 CAR T‐cell therapy experience in R/R GI lymphoma is still insufficient.

In our study, 12 patients with R/R GI lymphoma received anti‐CD19 CAR T‐cell therapy. We summarized the efficacy and side effects of these R/R GI lymphoma patients, especially the factors related to gastrointestinal bleeding in anti‐CD19 CAR T‐cell therapy, which was preliminarily analyzed. (ChiCTR1800019622).

## 2 PATIENTS AND METHODS

### 2.1 Patients enrolled in the study

This retrospective single‐center cohort study enrolled in 12 patients with relapsed/refractory (R/R) gastrointestinal (GI) B‐cell lymphoma who were admitted to our hospital between December 2018 and March 2022. All the 12 patients had gastrointestinal lesions confirmed by gastroenteroscopy at their first diagnosis and R/R status. These 12 patients with R/R GI lymphoma received anti‐CD19 CAR T‐cell therapy. None of the patients had undergone operation of gastrointestinal tract or bridging therapy before this study. The cut‐off date of our study was December 31, 2022. Follow‐up was carried out from the day of anti‐CD19 CAR T‐cell infusion to the cut‐off date or the death date.

### 2.2 Anti‐CD19‐CAR T cell therapy

All the 12 patients with R/R GI lymphoma received an anti‐CD19 CAR T‐cell infusion (*ChiCTR1800019622*). Anti‐CD19 CAR T‐cells in this clinical trial were CAR‐T cells of the second generation (4‐1BB co‐stimulating molecule, humanized CD19 CAR lentivirus, provided by Shanghai Gibe Company). All patients signed informed consent before therapy. This study was approved by the Ethics Committee of Tianjin First Central Hospital. Fludarabine (30 mg/m^2^/day) and cyclophosphamide (400 mg/m^2^/day) were administered from days −4 to −2 as lymphodepleting chemotherapy. The anti‐CD19 CAR T‐cell infusion dose was 2 × 10^6^ cells/kg on day 0 in all patients.

### 2.3 Response criteria and efficacy evaluation

One and 2 months after the CAR‐T cell infusion day, the efficacy was evaluated according to Lugano Revised Criteria.^19^ The primary endpoint of this study was the overall response rate (ORR), defined as the proportion of patients with a best overall response (including complete response [CR] and partial response [PR]). Stable disease (SD) and progression of disease (PD) were also accorded to Lugano Revised Criteria. Progression‐free survival (PFS) was calculated from the date of CAR‐T cell infusion to disease progression, death, or to the last date the patient was alive and without progression. Overall survival (OS) was calculated from the time of CAR‐T cell infusion until death or to the last date the patient was alive.

The response evaluation was detected using Positron Emission Tomography‐Computed Tomography (PET‐CT) or computed tomography (CT). Eight of the 12 patients with R/R GI lymphoma underwent gastroscopy and/or colonoscopy prior to anti‐CD19 CAR T‐cell therapy. The other four patients did not receive these evaluations due to disease progression factors. All patients, except those who died, underwent gastroscopy and/or colonoscopy after anti‐CD19 CAR T‐cell therapy to evaluate efficacy.

### 2.4 Patient grouping method

Bulky disease was defined as a tumor mass ≥7.5 cm in maximal diameter.^21^ To observe the efficacy and side effects of anti‐CD19 CAR T‐cell therapy for R/R GI lymphoma patients with different characteristics, we grouped patients with reference to literature report.^22^ We divided the 12 R/R B‐cell lymphoma patients into two groups based on the maximum tumor diameter: Bulky disease group (Gastrointestinal tumor mass ≥7.5 cm in maximal diameter) and NO bulky disease group (Gastrointestinal tumor mass <7.5 cm in maximal diameter). We divided them into two groups based on the site of gastrointestinal tract was involved: gastric involvement only group and Gastrointestinal involvement group (both might be involved in extra‐gastrointestinal involvement). We divided them into two groups based on the extra‐gastrointestinal involvement: gastrointestinal lesions only‐group and combined extra‐gastrointestinal lesions group. Then we divided them into two groups based on the characteristics of lymphoma: ulcer type group and lumps or nodules type group (determined by gastroenteroscopy). Finally, we divided them into two groups according to whether they had been bleeding in the previous treatment: gastrointestinal bleeding group and no gastrointestinal bleeding group.

### 2.5 The expression of anti‐CD19‐CAR T cells and cytokines

The transfection rate of anti‐CD19 CAR and the amplification rate of anti‐CD19 CAR T‐cells were determined using flow cytometry (FCM). During anti‐CD19 CAR T‐cell therapy, the expression of anti‐CD19‐CAR T cells was observed using FCM. Levels of interleukin‐6 (IL‐6) and interferon‐γ (IFN‐γ) were measured on days 0, 7, 14, 21, 28, and 60 in anti‐CD19‐CAR T‐cell therapy by enzyme‐linked immunosorbent assay (ELISA).

### 2.6 Adverse events (AEs)

In anti‐CD19 CAR T‐cell therapy, the grades of cytokine release syndrome (CRS) were evaluated based on the National Cancer Institute CTCAE v4.03,^23^ and the grades of neurotoxicity were evaluated based on ICANS.^24^

### 2.7 Statistical analysis

Data were expressed as mean ± standard deviation (SD), and differences between groups were compared using the log‐rank test. The nonparametric independent samples median test was used to compare median values between groups. The unpaired Student's *t*‐test was used to compare quantitative values between groups. Survival was analyzed with log‐rank test. Statistical analyses were performed using SPSS v24 and GraphPad Prism software (version 8.0). Statistical significance was set at two‐tailed *p* value < 0.05.

## 3 RESULTS

### 3.1 Baseline characteristics

Before anti‐CD19 CAR T‐cell therapy, baseline characteristics of these 12 patients with R/R GI lymphoma were listed in Table 1. In order to exclude active bleeding of lymphoma with gastrointestinal tract involvement before anti‐CD19 CAR T‐cell therapy, eight of the 12 patients with R/R GI lymphoma underwent gastroscopy and/or colonoscopy prior to this therapy.

**TABLE 1:** Baseline characteristics of the patients with R/R gastrointestinal lymphoma.

	Sex	Age	Subtype	Stage	IPI	Prelines of therapy	Poor prognosis	High tumor load	Gastric/Gastrointestinal involvement	Extra‐gastrointestinal lesions	GL lymphoma type
P 1	M	28	GCB	III	4	4	DE, TP53	Yes	Gastrointestinal	No	Lumps/nodules
P 2	F	45	Non‐GCB	IV	4	6	DH	Yes	Gastrointestinal	No	Lumps/nodules
P 3	F	58	GCB	IV	3	4	No	No	Gastric	No	Ulcer
P 4	M	72	tFL	IV	5	6	TP53	Yes	Gastric	Yes	Lumps/nodules
P 5	M	56	Non‐GCB	IV	4	5	DH	No	Gastrointestinal	Yes	Lumps/nodules
P 6	F	54	Non‐GCB	III	4	4	No	Yes	Gastrointestinal	Yes	Lumps/nodules
P 7	F	58	GCB	III	2	3	No	No	Gastric	No	Ulcer
P 8	F	62	Non‐GCB	IV	4	4	TP53	Yes	Gastric	Yes	Ulcer
P 9	F	70	Non‐GCB	IV	5	3	No	No	Gastric	No	Ulcer
P 10	M	42	GCB	IV	3	5	DE	No	Gastrointestinal	Yes	Ulcer
P 11	M	60	GCB	III	4	3	TP53	Yes	Gastrointestinal	No	Lumps/nodules
P 12	M	58	Burkitt	IV	3	3	TP53	No	Gastrointestinal	Yes	Lumps/nodules

Abbreviations: Burkitt, Burkitt's lymphoma; DE, Double expression; DH, Double Hit; F, Follicular lymphoma; GCB, Germinal center B‐cell‐like lymphoma; Non‐GCB, Non‐Germinal center B‐cell‐like lymphoma; tFL, FL develop histological transformation to DLBCL; TP53, TP53 is mutation or deletion.

### 3.2 Transduction efficiency of anti‐CD19 CAR and infusion dose

The mean transduction efficiency of anti‐CD19 CAR of all enrolled patients was 49.07 ± 8.37% on harvest date. All the 12 patients received 2.13 ± 0.45 × 10^6^ cells/kg autologous anti‐CD19 CAR T‐cell intravenously on infusion day.

### 3.3 Clinical responses to anti‐CD19 CAR T‐cell therapy

**FIGURE 1:**
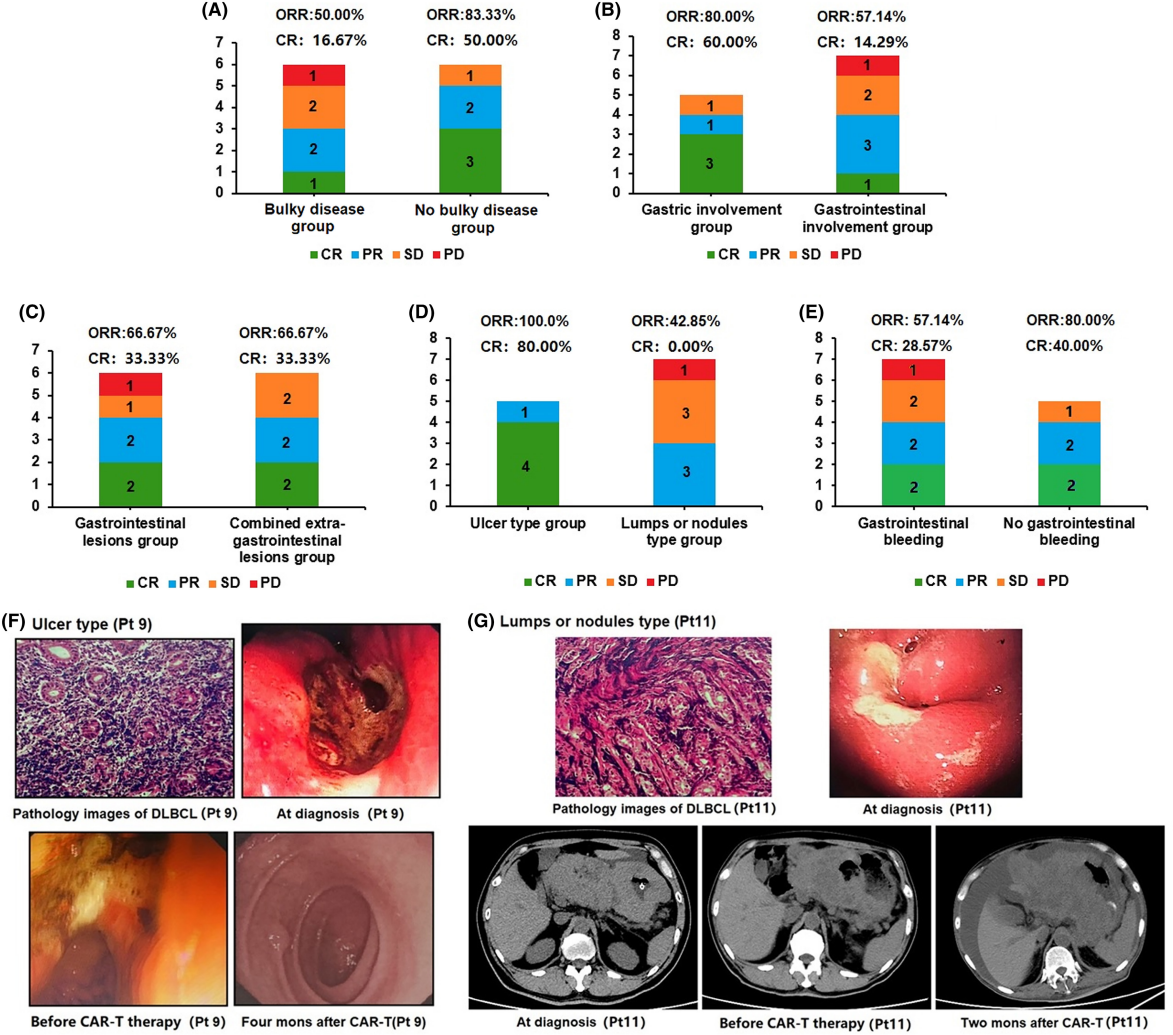
(A) ORR and CR rate in Bulky/No bulky disease group. (B) ORR and CR rate in gastric/Gastrointestinal involvement group. (C) ORR and CR rate in gastrointestinal/combined extra‐gastrointestinal lesions group. (D) ORR and CR rate in ulcer/lumps or nodules type group. (E) ORR and CR rate in with/without gastrointestinal bleeding group. (F) The pathology and gastroscopy results of Ulcer type. (G) The pathology and gastroscopy results of Lumps or nodules type.

The median follow‐up time was 9.9 months (range, 1.5–39.0 months). The objective response rate (ORR) was 66.67% (8/12) in these 12 patients. The ORR was 50.0% (3/6) in bulky disease group, while it was 83.33% (5/6) in no bulky disease group. The ORR was 80.00% (4/5) in gastric involvement group, while it was 57.14% (4/7) in gastrointestinal involvement group. The ORR was 66.67% (4/6) in gastrointestinal lesions‐only group, while it was 66.67% (4/6) in combined extra‐gastrointestinal lesions group. The ORR was 100.00% (5/5) in ulcer type group, while it was 42.85% (3/7) in lumps or nodules type group. Then the ORR was 57.14% (4/7) in gastrointestinal bleeding group, while it was 80.00% (4/5) in no gastrointestinal bleeding group (Figure 1A–E).

The CR rate was 33.33% (4/12) in these 12 patients. The CR was 16.67% (1/6) in bulky disease group, while it was 50.0% (3/6) in no bulky disease group. The CR was 60.00% (3/5) in gastric involvement group, while it was 14.29% (1/7) in gastrointestinal involvement group. The CR was 33.33% (2/6) in gastrointestinal lesions only‐group, while it was 33.33% (2/6) in combined extra‐gastrointestinal lesions group. The CR was 80.00% (4/5) in ulcer type group, while it was 0% (0/7) in lumps or nodules type group. The CR was 28.57% (2/7) in gastrointestinal bleeding group, while it was 40.00% (2/5) in no gastrointestinal bleeding group (Figure 1A–E).

The pathology and gastroscopy results of Pt 9, who obtained CR in her anti‐CD19 CAR T‐cell therapy, are shown in Figure 1F. The pathology, gastroscopy, and CT results of Pt 11 who obtained PD in his anti‐CD19 CAR T‐cell therapy are shown in Figure 1G.

### 3.4 Survival of the patients with R/R B‐cell lymphoma

**FIGURE 2:**
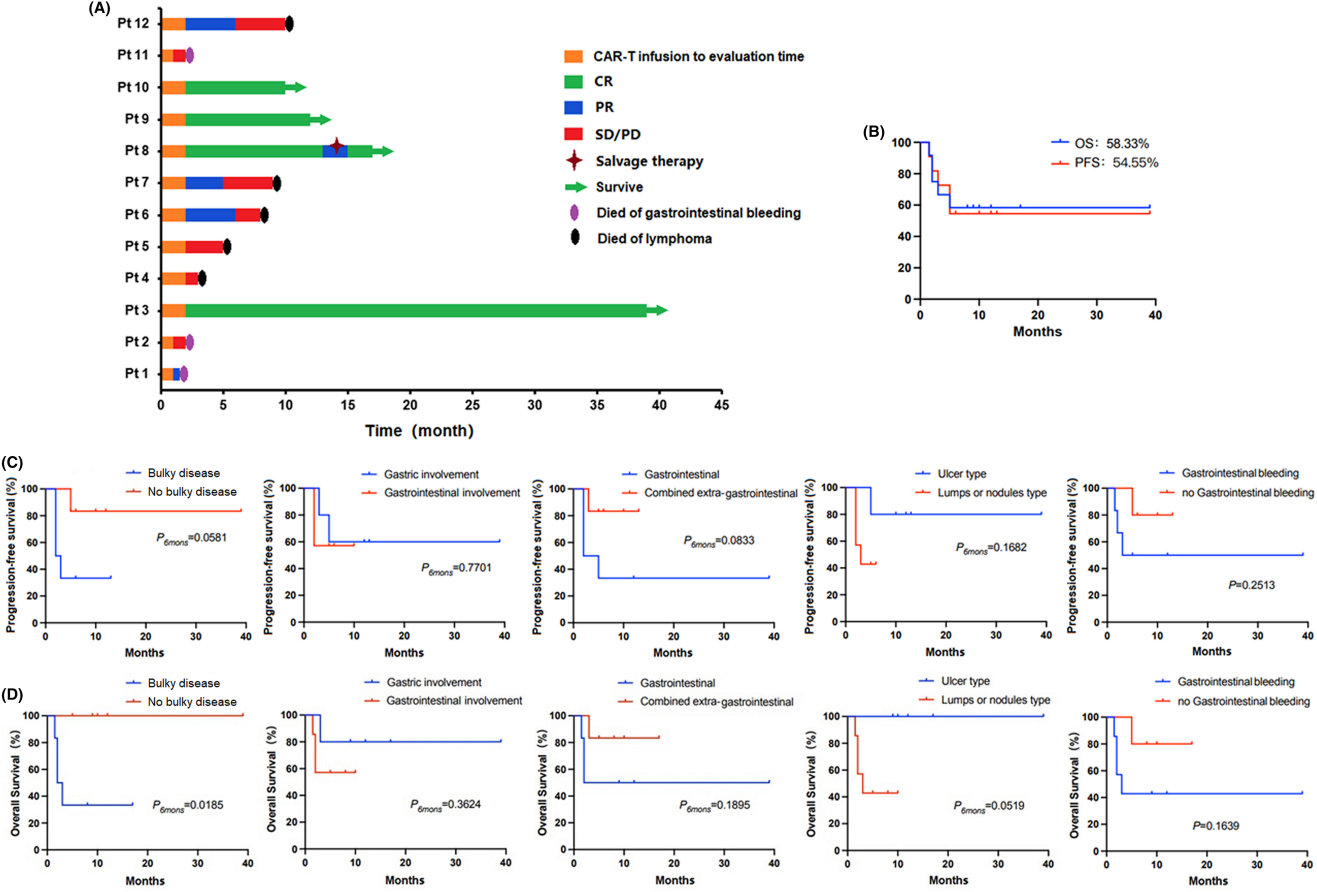
(A) The clinical response and prognosis of disease after anti‐CD19‐CAR T cell therapy of all the patients with R/R GI lymphoma. (B) The PFS and OS rate at 6 months after infusion of all the patients. (C, D) OS at 6 months was higher in no bulky disease group than that of in bulky disease group. There was no difference of the PFS or OS at 6 months between all the groups.

In our study, three of the four patients (Pt 3,9, and 10) who had obtained CR maintained durable remission until the cut‐off date. Only one patient (Pt 8) who had obtained CR developed disease progression later, then she obtained CR again after salvage therapy (programmed cell death 1 inhibitors). Three of the four patients (Pt 6,7, and 12) who had obtained PR developed disease progression again and then died. Pt 1 was evaluated as PR 1 month after CAR‐T cell infusion, but he died of intestinal perforation 1 month and a half after CAR‐T cell infusion. Pt 2 and Pt 11 were evaluated as SD and PD 1 month after CAR‐T cell infusion and soon died of intestinal perforation (Figure 2A).

For the 12 patients with R/R GI lymphoma, the PFS and OS rates at 6 months after infusion were 54.55% and 58.33%, respectively (Figure 2B).

There was no difference of the PFS at 6 months between the five groups (*p* = 0.0581, *p* = 0.7701, *p* = 0.0833, *p* = 0.1682, *p* = 0.2513; Figure 2C).

The OS at 6 months was higher in no bulky disease group than that of in bulky disease group (*p* = 0.0185). But there was no difference of the OS at 6 months between the other four groups (*p* = 0.3624, *p* = 0.1895, *p* = 0.0519, *p* = 0.1639; Figure 2D).

### 3.5 Anti‐CD19 CAR T‐cell amplification

**FIGURE 3:**
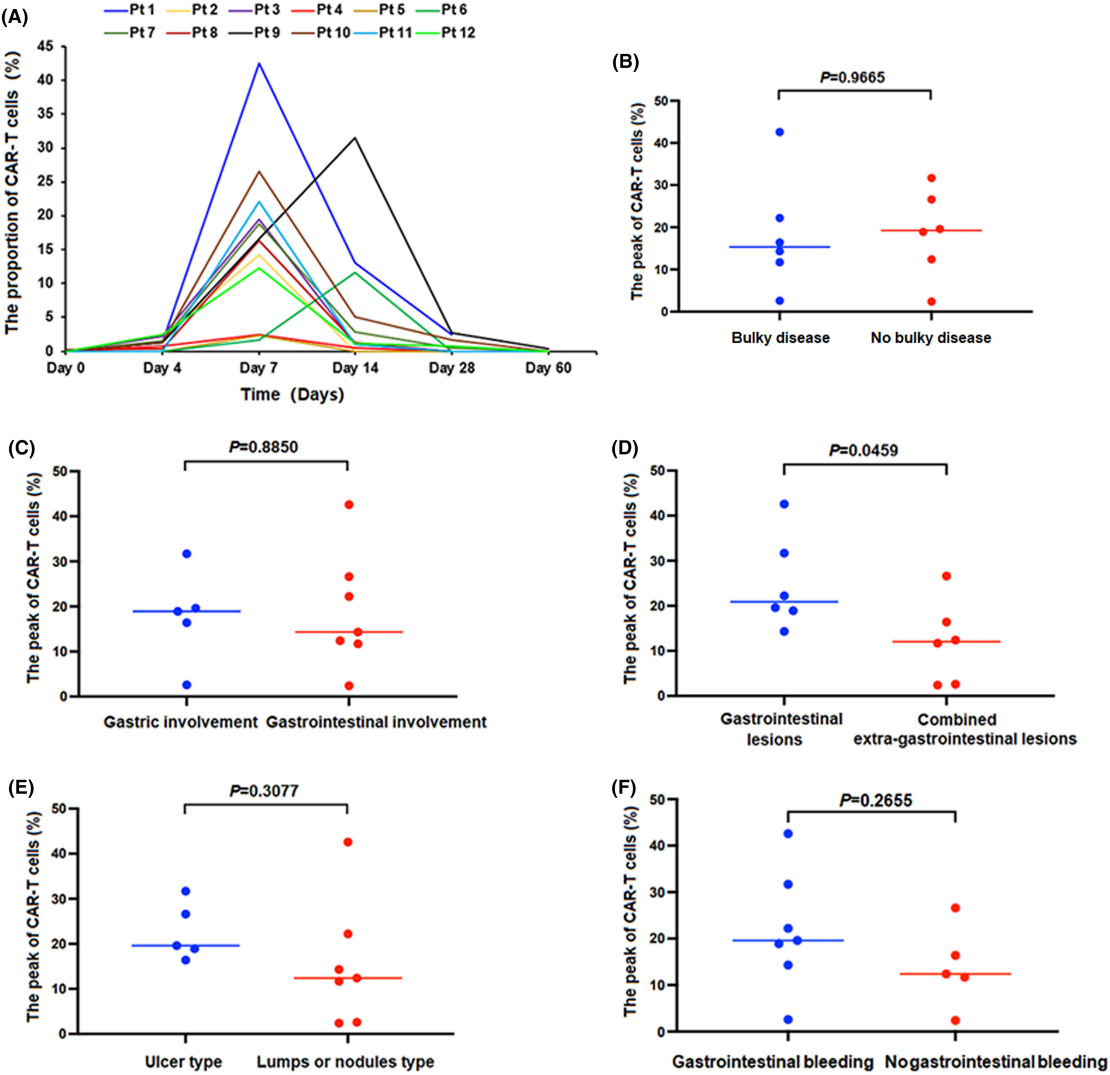
(A) Proportions of CAR‐T cells was observed after CAR‐T cell infusion. (B–F) There was no difference of the mean peak of anti‐CD19 CAR T‐cells between other groups except gastrointestinal lesions /combined extra‐gastrointestinal lesions group. (D) The mean peak of anti‐CD19 CAR T‐cells was higher in gastrointestinal lesions only group than that of in combined extra‐gastrointestinal lesions group.

In anti‐CD19 CAR T‐cell therapy, the proportion of anti‐CD19 CAR T‐cells in peripheral blood was detected 0, 7, 14, 28, and 60 days after CAR‐T cell infusion in all the 12 patients (Figure 3A).

The mean peak of anti‐CD19 CAR T‐cells was higher in gastrointestinal lesions‐only group than that of in combined extra‐gastrointestinal lesions group (*p* = 0.0459; Figure 3D). There was no difference of the mean peak of anti‐CD19 CAR T‐cells between the other four groups (*p* = 0.9665, *p* = 0.8850, *p* = 0.3077, *p* = 0.2655; Figure 3B,C,E,F).

### 3.6 Cytokine secretion in anti‐CD19 CAR T‐cell therapy

**FIGURE 4:**
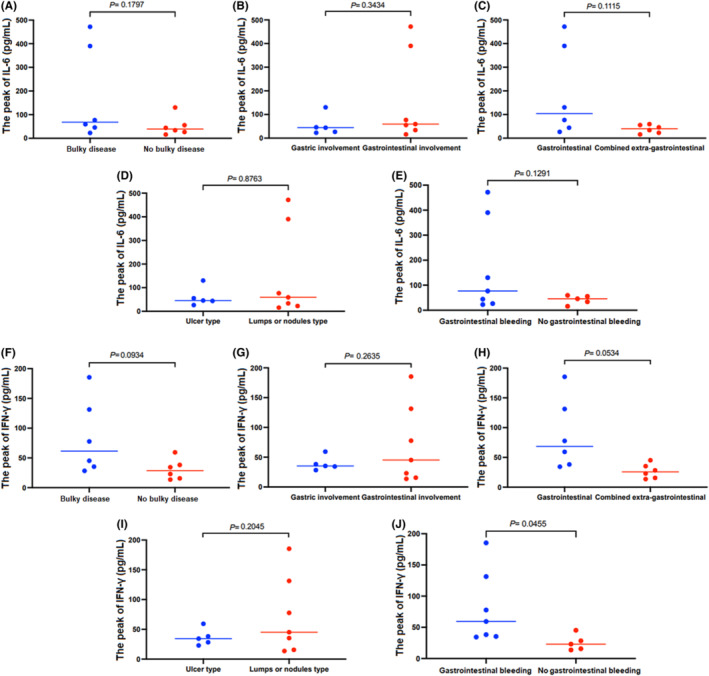
(A–E) There was no difference of the mean peak of IL‐6 between all the groups. (F–J) There was no difference of the mean peak of IFN‐γ between other groups except gastrointestinal bleeding /no gastrointestinal bleeding group.

The secretion of IL‐6 and IFN‐γ in anti‐CD19 CAR T‐cell therapy was detected 0, 7, 14, 28, and 60 days after CAR‐T cell infusion in all the 12 patients. There was no difference of the mean peak of IL‐6 between the five groups (*p* = 0.1797, *p* = 0.3434, *p* = 0.1115, *p* = 0.8763, and *p* = 0.1291; Figure 4A–E).

The mean peak of IFN‐γ was higher in gastrointestinal bleeding group than that of in no gastrointestinal bleeding group (*p* = 0.0455; Figure 4J). But there was no difference of the mean peak of IFN‐γ between the other four groups (*p* = 0.0934, *p* = 0.2635, *p* = 0.0534, and *p* = 0.2045; Figure 4F–I).

### 3.7 AEs in anti‐CD19 CAR T‐cell therapy

**FIGURE 5:**
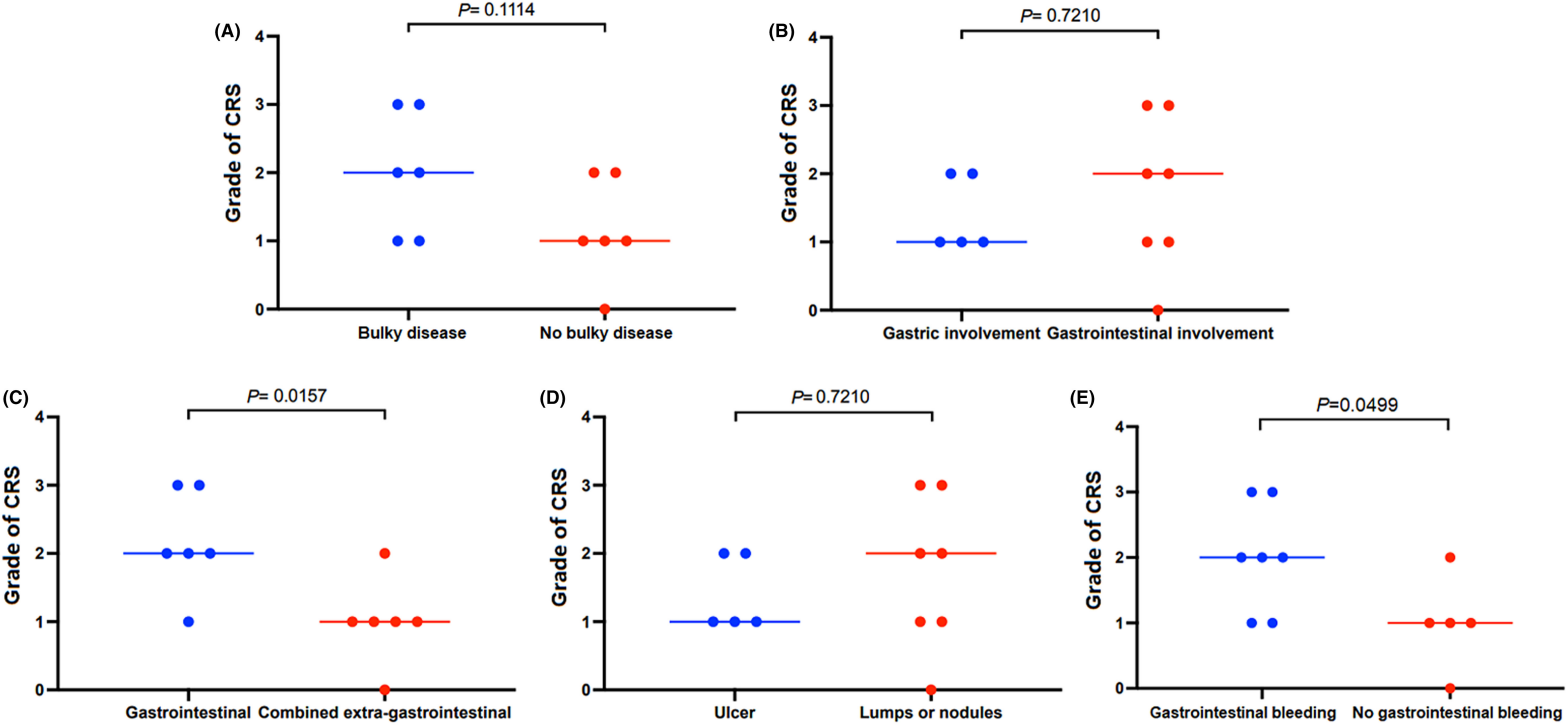
(A, B, D) There was no difference in the CRS grade between the groups. (C, E) The CRS grade was higher in gastrointestinal lesions group and in gastrointestinal bleeding group.

All the 12 patients in anti‐CD19 CAR T‐cell therapy recovered from their symptoms of AEs 6 to 21 days post‐CAR‐T cell infusion. The CRS grade was higher in gastrointestinal lesions‐only group than that of in combined extra‐gastrointestinal lesions group (*p* = 0.0157; Figure 5C), and higher in gastrointestinal bleeding group than that of in no gastrointestinal bleeding group (*p* = 0.0499; Figure 5 E). But there was no difference in the CRS grade between the other three groups (*p* = 0.1114, *p* = 0.7210, and *p* = 0.7210) (Figure 5 ABD). Only two patients (Pt 2 and Pt 7) developed grade 1 ICANS. Only the patients who were diagnosed with grade 3 CRS received tocilizumab and glucocorticoid. None of the 12 patients died of any level of CRS or ICANS during their anti‐CD19 CAR T‐cell therapy. And none of the patients died of bacterial infections; none of the patients were diagnosed with invasive fungal diseases.

Seven of the 12 patients (7/12, 58.33%) with R/R GI lymphoma developed gastrointestinal bleeding in their anti‐CD19 CAR T‐cell therapy. Three patients (Pt 1, Pt 2, and Pt 11) suffered from gastrointestinal perforation with peritonitis and gastrointestinal bleeding, which resulted in death after anti‐CD19‐CAR T‐cell therapy. Upper gastrointestinal bleeding occurred in three patients (Pt 3, Pt 7, and Pt 9), but they survived after anti‐CD19 CAR T‐cell therapy. Only one patient (Pt 4) developed lower gastrointestinal bleeding, but the patient did not die from it.

The characteristics of the patients with and without gastrointestinal bleeding are shown in Table 2. Incidence of gastrointestinal bleeding was higher in gastrointestinal lesions group than that of in combined extra‐gastrointestinal lesions group (*p* = 0.0152). Four out of six patients in group of gastrointestinal lesions group were patient with high tumor burden. There was no difference in ORR, CR, PFS, and OS at 6 months, PFS, and OS at 12 months in no gastrointestinal bleeding group and in gastrointestinal bleeding group. And there was no difference in the mean peak of anti‐CD19 CAR T‐cells, the proportion of ≥3 grade of CRS, or the proportion of ≥1 grade of ICANS in these two groups.

**TABLE 2:** Patient characteristics of gastrointestinal bleeding.

	Gastrointestinal bleeding (*n* = 7)	No gastrointestinal bleeding (*n* = 5)	*p* Value
Age (years)	55.86 ± 15.17	54.40 ± 7.54	0.8484
Sex (M/F)	3:4	3:2	1.0000^a^
IPI	3.86 ± 1.07	3.60 ± 0.55	0.6352
Prelines	4.14 ± 1.35	4.20 ± 0.84	0.9351
Tumor load			
High tumor load (%)	4/7 (57.14)	2/5 (40.00)	1.0000^a^
Low tumor load (%)	3/7 (42.86)	3/5 (60.00)	
Site of involvement			
Gastric involvement (%)	4/7 (57.14)	1/5 (20.00)	0.2929^a^
Gastrointestinal involvement (%)	3/7 (42.86)	4/5 (80.00)	
Extra‐gastrointestinal site			
No (%)	6/7 (85.71)	0/5 (0.00)	0.0152^a^
Yes (%)	1/7 (14.29)	5/5 (100.00)	
Type of GL			
Ulcer type (%)	3/7 (42.86)	2/5 (40.00)	1.0000^a^
Lumps or nodules type (%)	4/7 (57.14)	3/5 (60.00)	
ORR (%)	4/7 (57.14)	4/5 (80.00)	0.5758^a^
CR (%)	2/7 (28.57)	2/5 (40.00)	1.0000^a^
PFS at 6 months (%)	2/7 (28.57)	4/5 (80.00)	0.2424^a^
OS at 6 months (%)	3/7 (42.86)	4/5 (80.00)	0.2929^a^
PFS at 12 months (%)	2/7 (28.57)	1/5 (20.00)	1.0000^a^
OS at 12 months (%)	2/7 (28.57) 21.70 ± 12.71	1/5 (20.00) 13.90 ± 8.76	1.0000^a^
CAR‐T cell peak (%)	2/7 (28.57)	0/5 (0.00)	0.2655
CRS (≥3 grade)	2/7 (28.57)	0/5 (0.00)	0.4697^a^
ICANS (≥1 grade)	3/7 (42.86)	0/5 (0.00)	0.4697^a^
Died in CAR‐T therapy (%)			0.2045^a^

^a^ Fisher exact probability method.

## 4 DISCUSSION

Although anti‐CD19 CAR T‐cell therapy has shown a very effective salvage strategy in R/R B‐cell lymphomas,^17^, ^18^, ^19^, ^20^ anti‐CD19 CAR T‐cell therapy in R/R GI lymphoma has been poorly reported, and experience needs to be summarized. GI lymphoma carries the risk of perforation and bleeding, especially in patients with R/R GI lymphoma.^8^, ^25^ Because these patients were often excluded from many anti‐CD19 CAR T‐cell therapy clinical trials, safety and efficacy data for this therapy are lacking. In our study, we explored and summarized the efficacy and side effects of anti‐CD19 CAR T‐cell therapy in patients with R/R GI lymphoma based on the different characteristics of GI lymphoma. These characteristics of GI lymphoma including: Bulky/No bulky disease, Gastric/Gastrointestinal involvement, Gastrointestinal/Combined extra‐gastrointestinal lesions, Ulcer/Lumps or nodules type, With/without gastrointestinal bleeding.

The ORR was 66.67% and the CR rate was 33.33% in our study of anti‐CD19 CAR T‐cell therapy in patients with R/R GI lymphoma. Compared with the two large clinical trials of anti‐CD19 CAR T‐cell therapy to R/R B‐cell lymphoma, ZUMA‐1 and JULIET,^17^, ^18^ the ORR and CR in our study were lower than that the in ZUMA‐1 (82% and 54%) and in JULIET (52% and 40%). In a study of anti‐CD22/CD19 CAR T‐cell therapy in R/R GI lymphoma, the ORR was 71% (10/14) and CR was 50% (7/14).^26^ In other study of anti‐CD19 CAR T‐cell therapy in R/R GI lymphoma, the ORR was 63% and CR was 42%.^27^ There was no significant difference in ORR, PFS, and OS between patients with GI involvement and those without GI involvement. The outcomes of patients with GI involvement before CAR‐T cell therapy are similar to those without GI involvement.^27^ Our study focused on the characteristics of patients with R/R GI lymphoma receiving anti‐CD19 CAR T‐cell therapy. In our study, except the ORR and CR results of Gastrointestinal/Combined extra‐gastrointestinal lesions group were similar, the ORR and CR showed differences in other groups of characteristics. The ORR and CR of no bulky disease group, gastric involvement only, ulcer type, and no gastrointestinal bleeding group were higher, while the ORR and CR of bulky disease, gastrointestinal involvement, lumps or nodules type, and gastrointestinal bleeding group were lower.

For the 12 R/R GI lymphoma patients, the PFS and OS rate at 6 months were 54.55% and 58.33%, respectively in our study. The PFS and OS in anti‐CD22/CD19 CAR T‐cell therapy study to R/R GI lymphoma patients were 71.4% and 50.0%, respectively.^26^ The outcomes of R/R lymphoma patients with GI were similar to those without GI involvement in another such study.^27^ In our study, except that the OS at 6 months in no bulky disease group was higher than that in bulky disease group, the results of PFS or OS in other characteristic groups were similar.

The incidence rates of severe CRS and ICANS, length of hospital stay, and use of tocilizumab and glucocorticoid were similar in patients with GI involvement and patients without GI involvement.^27^ In another study, CRS and gastrointestinal adverse events were generally mild and manageable.^26^ In our study, the side effects of anti‐CD19 CAR T‐cell therapy in R/R GI lymphoma were analyzed. The mean peak of anti‐CD19 CAR T‐cells was higher in gastrointestinal lesions‐only group, while the mean peak of IFN‐γ was higher in gastrointestinal bleeding group. But there was no difference between the mean peak of anti‐CD19 CAR T‐cells and the mean peak of IL‐6 and IFN‐γ in other groups. Then the CRS grade was higher in gastrointestinal lesions group and in gastrointestinal bleeding group. Therefore, the severe side effects of anti‐CD19 CAR T‐cell therapy were higher in patients with gastrointestinal lesions and gastrointestinal bleeding.

## 5 CONCLUSION

In conclusion, we demonstrated that the ORR and CR of bulky disease group, gastrointestinal involvement, lumps or nodules type, and gastrointestinal bleeding group were lower. The CRS grade was higher in gastrointestinal lesions group and in gastrointestinal bleeding group. Patients with gastrointestinal involvement only were at higher risk for gastrointestinal bleeding.

## AUTHOR CONTRIBUTIONS

**Yi Li Jiang:** Conceptualization (equal); data curation (equal); formal analysis (equal); investigation (equal); methodology (equal); project administration (equal); resources (equal); software (equal); writing – original draft (equal); writing – review and editing (equal). **Juan Mu:** Data curation (equal); software (equal). **Rui Cui:** Validation (equal). **Xin Li:** Resources (equal). **Jia Wang:** Resources (equal). **Qing Li:** Resources (equal). **Jingyi Li:** Supervision (equal). **Nan Mou:** Resources (equal). **Qi Deng:** Conceptualization (equal); data curation (equal); formal analysis (equal); investigation (equal); methodology (equal); project administration (equal); software (equal); supervision (equal); validation (equal); visualization (equal); writing – original draft (equal); writing – review and editing (equal).

## FUNDING INFORMATION

This work was supported by the Sponsored by Tianjin Health Research Project (TJWJ2022XK021 and TJWJ2023ZD003). Chinese Society of Clinical Oncology Beijing Xisike Clinical Oncology Research Foundation (Y‐SY2021QN‐0184, Y‐Young2022‐0209, and Y‐NCJH202201‐0027).

## CONFLICT OF INTEREST STATEMENT

The authors declare that the research was conducted in the absence of any commercial or financial relationships that could be construed as a potential conflict of interest.

## ETHICS STATEMENT

This study was approved by the Medical Ethics Committee of the Department of Hematology, Tianjin First Center Hospital (Tianjin, China). (Approved No. of ethic committee: 2018N105KY). The patient gave their written informed consent in accordance with the Declaration of Helsinki. The Clinical trial in our study was registered at ChiCTR1800019622.

## Data Availability

The datasets used and/or analyzed during the current study are available from the corresponding author on reasonable request.

## References

[1] Peng JC , Zhong L , Ran ZH . Primary lymphomas in the gastrointestinal tract. J Dig Dis. 2015;16(4):169‐176. doi:10.1111/1751-2980.12234 25678011

[2] Siegel RL , Miller KD , Jemal A . Cancer statistics, 2019. CA Cancer J Clin. 2019;69(1):7‐34. doi:10.3322/caac.21551 30620402

[3] Yin X , Xu A , Fan F , et al. Incidence and mortality trends and risk prediction nomogram for extranodal diffuse large B‐cell lymphoma: an analysis of the surveillance, epidemiology, and end results database. Front Oncol. 2019;9:1198. doi:10.3389/fonc.2019.01198 31781500 PMC6861389

[4] d'Amore F , Brincker H , Gronbaek K , et al. Non‐Hodgkin's lymphoma of the gastrointestinal tract: a population‐based analysis of incidence, geographic distribution, clinicopathologic presentation features, and prognosis. Danish lymphoma Study group. J Clin Oncol. 1994;12(8):1673‐1684. doi:10.1200/JCO.1994.12.8.1673 8040680

[5] Lightner AL , Shannon E , Gibbons MM , Russell MM . Primary gastrointestinal non‐Hodgkin's lymphoma of the small and large intestines: a systematic review. J Gastrointest Surg. 2016;20(4):827‐839. doi:10.1007/s11605-015-3052-4 26676930

[6] Daum S , Ullrich R , Heise W , et al. Intestinal non‐Hodgkin's lymphoma: a multicenter prospective clinical study from the German Study group on intestinal non‐Hodgkin's lymphoma. J Clin Oncol. 2003;21(14):2740‐2746. doi:10.1200/JCO.2003.06.026 12860953

[7] Nakamura S , Matsumoto T , Takeshita M , et al. A clinicopathologic study of primary small intestine lymphoma: prognostic significance of mucosa‐associated lymphoid tissue‐derived lymphoma. Cancer. 2000;88(2):286‐294. doi:10.1002/(sici)1097-0142(20000115)88:2<286::aid-cncr7>3.0.co;2-z 10640959

[8] Vaidya R , Habermann TM , Donohue JH , et al. Bowel perforation in intestinal lymphoma: incidence and clinical features. Ann Oncol. 2013;24(9):2439‐2443. doi:10.1093/annonc/mdt188 23704194 PMC3755328

[9] Koch P , del Valle F , Berdel WE , et al. Primary gastrointestinal non‐Hodgkin's lymphoma: II. Combined surgical and conservative or conservative management only in localized gastric lymphoma–results of the prospective German Multicenter Study GIT NHL 01/92.J. Clin Oncol. 2001;19(18):3874‐3883. doi:10.1200/JCO.2001.19.18.3874 11559725

[10] Gou HF , Zang J , Jiang M , Yang Y , Cao D , Chen XC . Clinical prognostic analysis of 116 patients with primary intestinal non‐Hodgkin lymphoma. Med Oncol. 2012;29(1):227‐234. doi:10.1007/s12032-010-9783-x 21193968

[11] Shen Y , Ou J , Wang B , Wang L , Xu J , Cen X . Influence of severe gastrointestinal complications in primary gastrointestinal diffuse large B‐cell lymphoma. Cancer Manag Res. 2021;13:1041‐1052. doi:10.2147/CMAR.S295671 33568947 PMC7869708

[12] Sehn LH , Donaldson J , Chhanabhai M , et al. Introduction of combined CHOP plus rituximab therapy dramatically improved outcome of diffuse large B‐cell lymphoma in British Columbia. J Clin Oncol. 2005;23(22):5027‐5033. doi:10.1200/JCO.2005.09.137 15955905

[13] Abbott S , Nikolousis E , Badger I . Intestinal lymphoma – a review of the management of emergency presentations to the general surgeon. Int J Colorectal Dis. 2015;30(2):151‐157. doi:10.1007/s00384-014-2061-1 25374417

[14] Kim SJ , Kang HJ , Kim JS , et al. Comparison of treatment strategies for patients with intestinal diffuse large B‐cell lymphoma: surgical resection followed by chemotherapy versus chemotherapy alone. Blood. 2011;117(6):1958‐1965. doi:10.1182/blood-2010-06-288480 21148334

[15] Iida T , Nozawa H , Sonoda H , et al. Upfront surgery for small intestinal non‐Hodgkin's lymphoma. Anticancer Res. 2020;40(4):2373‐2377. doi:10.21873/anticanres.14206 32234940

[16] Hong YW , Kuo IM , Liu YY , Yeh TS . The role of surgical management in primary small bowel lymphoma: a single‐center experience. Eur J Surg Oncol. 2017;43(10):1886‐1893. doi:10.1016/j.ejso.2017.06.016 28751057

[17] Neelapu SS , Locke FL , Bartlett NL , et al. Axicabtagene ciloleucel CAR T‐cell therapy in refractory large B‐cell lymphoma. N Engl J Med. 2017;377(26):2531‐2544. doi:10.1056/NEJMoa1707447 29226797 PMC5882485

[18] Schuster SJ , Bishop MR , Tam CS , et al. Tisagenlecleucel in adult relapsed or refractory diffuse large B‐cell Lymphoma. N Engl J Med. 2019;380(1):45‐56. doi:10.1056/NEJMoa1804980 30501490

[19] Abramson JS , Palomba ML , Gordon LI , et al. Lisocabtagene maraleucel for patients with relapsed or refractory large B‐cell lymphomas (TRANSCEND NHL 001): a multicentre seamless design study. Lancet. 2020;396(10254):839‐852. doi:10.1016/S0140-6736(20)31366-0 32888407

[20] Wang M , Munoz J , Goy A , et al. KTE‐X19 CAR T‐cell therapy in relapsed or refractory mantle‐cell lymphoma. N Engl J Med. 2020;382(14):1331‐1342. doi:10.1056/NEJMoa1914347 32242358 PMC7731441

[21] Network N C C . Clinical practice guidelines in oncology. B‐Cell Lymphomas. Version 2. 2019. https://www.nccn.org/professionals/physician_gls/pdf/b‐cell

[22] Castellino A , Tun AM , Wang Y , et al. Clinical characteristics and outcomes of primary versus secondary gastrointestinal mantle cell lymphoma. Blood Cancer J. 2021;11(1):8. doi:10.1038/s41408-020-00394-z 33414416 PMC7791108

[23] NIH . Common terminology criteria for adverse events (CTCAE). 2017. https://ctep.cancer.gov/protocoldevelopment/electronic_applications/docs/CTCAE_v5_Quick_Reference_8.5x11

[24] Lee DW , Santomasso BD , Locke FL , et al. ASTCT consensus grading for cytokine release syndrome and neurologic toxicity associated with immune effector cells. Biol Blood Marrow Transplant. 2019;25(4):625‐638. doi:10.1016/j.bbmt.2018.12.758 30592986 PMC12180426

[25] Sabattini E , Bacci F , Sagramoso C , Pileri SA . WHO classification of tumours of haematopoietic and lymphoid tissues in 2008: an overview. Pathologica. 2010;102(3):83‐87. 21171509

[26] Zeng C , Cheng J , Li T , et al. Efficacy and toxicity for CD22/CD19 chimeric antigen receptor T‐cell therapy in patients with relapsed/refractory aggressive B‐cell lymphoma involving the gastrointestinal tract. Cytotherapy. 2020;22(3):166‐171. doi:10.1016/j.jcyt.2020.01.008 32063474

[27] Cortes‐Bullich A , Perez A , Bachmeier C , et al. Outcomes of CD19 chimeric antigen receptor T cell therapy in patients with gastrointestinal tract involvement of large B cell lymphoma. Transplant Cell Ther. 2021;27(9):768.e1‐768.e6. doi:10.1016/j.jtct.2021.05.018 PMC8403629 34077811

